# The Dental Aspect of It

**Published:** 1892-12

**Authors:** A. Bethune Patterson


### THE DENTAL ASPECT OF IT.

BY A. BETHUNE PATTERSON, M.D.[1]

In the June issue of the *Dental Cosmos* there is an editorial on the use of the lancet during first dentition. The writer labors to show the importance of more general information upon the conditions which call for the use of the lancet in teething, and deplores the ignorance manifested by medical men in anatomy, physiology, histology and pathology; and also in the etiology of disease, especially as it bears upon pathological conditions found in the jaws of teething children, which conditions, to the editor's mind, are responsible for many, if not all, of the diseases of infantile life.

He would force practitioners to accept his views by placing them before the profession as facts. He says in reference to the therapeutic value of the lancet, "this is simply a question of fact, regardless of any theories involved."

With this flood of light from the dental side, it is presumed that dentists and medical practitioners will supply themselves at once with gum-lancets, the panacea of all the ills of infantile life, or remain "culpable" and "ignorant" obstructionists to modern therapeutics. The editor continues: "Gum-lancing is a therapeutic measure of unquestionable value, relieving the pulp-irritation set up by backward pressure of the developing root. . . . But why, in the light of all that has been written on the subject from a dental stand-point, should any be ignorant? . . . Doubly culpable is a writer

---

[1] Late Assistant to the Royal London Ophthalmic Hospital, and Golden Square Throat Hospital, London, England.

of acknowledged authority who condemns the operation of gum-lancing as a therapeutic measure."

The author referred to is F. Forchheimer, M.D., Professor of Physiology and Clinical Diseases of Children in the Medical College of Ohio, and a member of the Association of American Physicians and also of the American Pediatric Society, etc. This author condemns gum-lancing, and the editor of the *Dental Cosmos* "records a protest against the fallacies here promulgated, in the hope of preventing the serious consequences which must necessarily follow such antiquated teaching, an adherence to which is largely responsible for the high rate of infantile mortality." Exactly how our learned critic arrived at these remarkable conclusions and the "facts" is of great interest to practitioners of scientific medicine. Sections of the tissue in question properly investigated under the microscope reveal the only solution to the problem. And by this means only can a full explanation be given of that much-dreaded condition characterized by "unyielding and overlying gum-tissue." Hypertrophy and atrophy, and in fact all other morbid conditions of the cellular tissue, nerves, blood-vessels, can be understood only by aid of the microscope. It is to be inferred that the editor has not entered very far into the study of pathological dentition, for he says, "There may be produced a hyperæmia sufficient possibly to produce the protrusion of a part of the mass from the incomplete aperture of the root, giving abundant cause for extreme constitutional disturbance." This does not show that the editor is very confident in the position he has assumed. Again, we are informed that "in the adult, irritation of a dental nerve may give rise to otalgia, otorrhœa, deafness, amaurosis, hemicrania, neuralgia, hysteria, chorea, epilepsy, tetanus, and it is not only possible, but highly probable, that a like irritation may be the occasion of grave and even fatal disorders in the infant." What proof have we of such statements? Such conclusions would have claimed precedence some twenty years ago. But the great hurricane of progressive medicine has swept away such wild and speculative theories. I hold that it is not in good taste, neither is it fair, to brand practitioners and "authors of high authority" with deplorable ignorance, and especially is this out of taste in an editor who guards his opinions and expressions with such terms as "may be," "possibly," "highly probable," etc. Such opinions the editor expects medical men to accept as a lucid explanation of the pathological condition which in his mind is responsible for the present high rate of infantile mortality.

It is upon a clinical and scientific stand-point, keeping before me the laws of modern pathology, that I venture to take issue with the learned editor. Research in the etiology of disease has opened the way to a clear understanding of at least some of the pathological conditions. The investigations have led us into the field of bacteriology. By the study of micro-organisms we have accounted for many phenomena which before could only be speculated upon.

In considering gum-lancing, the question of inflammation cannot be ignored, neither the so-called "reflex neurosis,"—a bulwark behind which a vast amount of ignorance takes refuge.

To enter into a discussion of a number of diseases that are daily sending the little ones to an untimely grave is not the object of this paper. A brief reference to only a few of the fatal diseases of children will prove that pressure upon formative pulp cannot be the "occasion of grave and even fatal disorders in the infant."

Mothers and doctors have been bothering themselves for ages about nature's way of getting out teeth. Nearly all the morbid conditions of this period have been traced to this physiological process. Swollen and inflamed gums have attracted the attention of the illiterate as well as the educated. So common is this pathological condition, accompanied by grave enteric and nervous disturbances, that they have been readily associated as cause and effect, and many have been the arguments knit together to satisfactorily demonstrate the plausibility of the theory.

Medical men are agreed as to the etiology of inflammation. This pathological condition has its origin in micro-organisms. It would be a waste of time, too, in the light of what has been developed from a medical stand-point, to offer proof of this statement. Swollen and inflamed gums are not alluded to in the editorial as characterizing "pathological dentition," and as these signs are the only evidence of a morbid condition it is necessary that they be carefully considered. There are two ways by which bacteria find their way into the tissue in question,—through the circulation and through the buccal cavity, which cavity is the habitat of several different species of bacteria. While some of them appear to be harmless, others are inflammatory. The latter find their way into the gum-tissue by accidents which are traumatic in character. The disposition of an infant to bite on any hard substance that it can get in its mouth is due, in my opinion, to the itching sensation which results from external pressure compressing the nerves supplying the intervening and absorbing structures. This is an invariable and constant condition. Mothers and nurses, eager to supply every apparent comfort and whim, have confidence in rubber rings, knife- and tooth brush-handles, friction and pressure upon the overlying gum, as a means of assisting the exit of the teeth. Thus supplied with the would-be auxiliaries, the infant, unmindful of the consequences, comes down with too much force, with the result of a rupture of the epithelium and underlying tissues. Fissures are thus formed through which pyogenic germs find their way into the underlying structure, the cells of which have already been crippled by the traumatism. At once the operation of development commences which is signalled by the common evidences of inflammation,—viz., redness and swelling, etc. Another way by which inflammation is frequently set up is as follows: In the cutting of a molar, one of the cusps makes its appearance before any of the others, leaving an overlying flap which forms a pocket for the reception of particles of food, accompanied by inflammatory germs which here find conditions favorable to development. The result is inflammation. This condition calls for a lancet, the indications being those of an abscess. The resulting hemorrhage would wash out the bacteria and foreign matter, and relief would follow.

The editor, strong in his faith in "the light that has been developed on the dental side of this question," is uncompromising in his belief that "the present high rate of infant mortality is due to the irritation of the dental nerve set up by backward pressure upon the formative pulp." He is confident that his learned colleague—Dr. J. W. White—has proven conclusively that "in the adult the irritation of a dental nerve may give rise to otalgia, otorrhœa, deafness, amaurosis, hemicrania, neuralgia, hysteria, chorea, epilepsy, tetanus," etc. Dr. J. W. White concludes: "It is surely not only possible, but highly probable, that a like irritation may be the occasion of grave and even fatal disorders in the infant."

Reasoning from this stand-point, it would be logical to conclude that the gratification of the infant to bite on hard substances would increase the backward pressure, thus increasing the "distressing symptoms," and the diseases incident to first dentition, especially fits, diarrhœa, cholera morbus, etc., would be intensified.

In the study of the cause of disease at the present day we find bacteriology and pathology moving along hand in hand dispensing light, the rays of which are daily penetrating the hovel as well as the palace, carrying comfort and good cheer. The observations of pure cultures of micro-organisms in the tissues of animals, as well as in the tissue of human beings, have demonstrated conclusively the part they play in the cause of disease. So no doubt remains in the minds of medical men as to the cause of tetanus, cholera morbus, and the various forms of diarrhœa. Bacteria of a well-known shape, having color which is reflected in the characteristic green stools of teething infants, find their way into the alimentary tract and are responsible for the enteric troubles which help to swell the high rate of infant mortality.

Further comment upon modern etiology would be akin to supererogation, and, as a parting word to our dental editor, must say we will regard with reverence the "light" that has been developed on the dental side of the issue as a beacon, warning us of dangerous ground upon which the fondest hopes have been wrecked, and to say we are now headed for that revolving light in mid-ocean of progressive medicine, a field which, when diligently cultivated, yields a harvest rich in good to suffering humanity.

